# Involvement of Autophagic Pathway in the Progression of Retinal Degeneration in a Mouse Model of Diabetes

**DOI:** 10.3389/fncel.2016.00042

**Published:** 2016-02-19

**Authors:** Ilaria Piano, Elena Novelli, Luca Della Santina, Enrica Strettoi, Luigi Cervetto, Claudia Gargini

**Affiliations:** ^1^Department of Pharmacy, University of PisaPisa, Italy; ^2^National Research Council (CNR), Neuroscience InstitutePisa, Italy

**Keywords:** diabetic retinopathy, mouse model, retinal damage, photoreceptors, autophagy

## Abstract

The notion that diabetic retinopathy (DR) is essentially a micro-vascular disease has been recently challenged by studies reporting that vascular changes are preceded by signs of damage and loss of retinal neurons. As to the mode by which neuronal death occurs, the evidence that apoptosis is the main cause of neuronal loss is far from compelling. The objective of this study was to investigate these controversies in a mouse model of streptozotocin (STZ) induced diabetes. Starting from 8 weeks after diabetes induction there was loss of rod but not of cone photoreceptors, together with reduced thickness of the outer and inner synaptic layers. Correspondingly, rhodopsin expression was downregulated and the scotopic electroretinogram (ERG) is suppressed. In contrast, cone opsin expression and photopic ERG response were not affected. Suppression of the scotopic ERG preceded morphological changes as well as any detectable sign of vascular alteration. Only sparse apoptotic figures were detected by terminal deoxynucleotidyl transferase dUTP nick end labeling (TUNEL) assay and glia was not activated. The physiological autophagy flow was altered instead, as seen by increased LC3 immunostaining at the level of outer plexiform layer (OPL) and upregulation of the autophagic proteins Beclin-1 and Atg5. Collectively, our results show that the streptozotocin induced DR in mouse initiates with a functional loss of the rod visual pathway. The pathogenic pathways leading to cell death develop with the initial dysregulation of autophagy well before the appearance of signs of vascular damage and without strong involvement of apoptosis.

## Introduction

Chronic exposure to hyperglycemia triggers a chain of biochemical and functional processes leading to vascular damage and to a series of central and peripheral neuropathies. The diabetic retinopathy (DR) is the one of the most common complications of diabetes (Antonetti et al., 2006; Kaul et al., 2013; Blake and Trounce, 2014) and a leading cause of blindness worldwide (Cheung et al., 2010). The notion that DR is essentially a microvascular disease has been recently challenged by several studies reporting that the vascular changes are preceded by signs of functional impairment and loss of neural retinal cells (Zeng et al., 2000; Martin et al., 2004; Bearse et al., 2006; Gaucher et al., 2007). Furthermore, the mechanisms of photoreceptors death have not been conclusively established. This lack of understanding has seriously limited the therapeutic options available for the ophthalmologist and there is a need to identify the pathways that initiate retinal cell damage and drive progression to overt retinopathy. Apoptotic pathways are involved in the death of ganglion cells (Martin et al., 2004; Barber et al., 2005), while cell death in the outer retina remains unexplained. Apoptosis in diabetes was observed in human retinas as well as in animal models of DR (Abu-El-Asrar et al., 2004; Barber et al., 2005), causing retinal thinning in rats starting 6 months after diabetes induction (Park et al., 2003). It must be pointed out, however, that early changes in outer retinal neurons occur well before the appearance of apoptotic figures (Énzsöly et al., 2014), with evidence supporting a role for autophagy in the pathophysiology of type-1 diabetes from a variety of tissues (Gonzalez et al., 2011).

The objective of this study was to investigate the sequence of changes leading to retinal damage in a mouse model of type-1 streptozotocin (STZ) induced diabetes (Jo et al., 2013). Our results showed that specific changes in function, morphology and biochemistry develop within 12 weeks after diabetes induction, consisting in the suppression of scotopic electroretinogram (ERG) response, rods loss and reduction of retinal thickness. All these changes preceded any detectable vascular alteration, apoptotic figures were rare and glia did not activate. On the other hand, specific tests reveal a marked up-regulation of autophagic processes suggesting that the autophagic pathway is involved in the initial damage of the visual neurons of the rod pathway.

## Materials and Methods

### Animals

C57BL/6J mice (Jackson Laboratories, Sacramento, CA, USA), were housed in 12 h light/dark cycle with illumination levels below 60 lux. Diabetes was induced at P30 by single i.p. injection of 150 mg/Kg STZ, (Sigma-Aldrich, St. Louis, MO, USA; Wang et al., 2010). The blood glucose concentration was measured before each experiment from the tail vein using a One Touch Ultra Easy device (LifeScan, Roma, Italy). Hyperglycemia was defined as blood glucose >16.7 mmol/L, 48 h after STZ injection. Age-matched control mice received a single i.p. injection of vehicle (citrate buffer, pH 4.5). Mice were no treated with insulin. All mice were then tested at 4, 8 and 12 weeks post-injection.

All experiments were carried out in accordance to the ARVO statement for the use of animals in Ophthalmic and Vision Research. Animal protocol was approved by the Animal Care Committee of the University of Pisa, Italy (Protocol. N.4886, April 4th, 2011). For all procedures, mice were anesthetized with Urethane 20% (100 μl/10 g, Sigma-Aldrich).

### Immunohistochemistry

Retinal section preparation has been previously described in detail (Della Santina et al., 2012). Sections were washed 3 × 10 min in phosphate buffered saline (PBS), then incubated 45 min in blocking solution (1% bovine serum albumin; BSA, 0.3% Triton-X100 in PBS). Sections were incubated overnight at 4°C with primary antibodies (Table 1). Sections were then washed 3 × 10 min in PBS and incubated with secondary antibodies (Table 1) for 2 h at room temperature. Nuclear staining was obtained with propidium iodide. Confocal images were obtained with a Leica TCS-SL microscope (Leica Microsystem, Wetzlar, Germany).

**Table 1:** **List of primary and secondary antibodies**.

	Host	Dilution	Supply	Application
Primary antibody
Protein Kinase C α	Mouse	1:100	Sigma-Aldrich	IF*
PSD95	Mouse	1:1000	Millipore	IF*
GFAP	Rabbit	1:100	Sigma-Aldrich	IF*
LC3A	Rabbit	1:200	Cell Signaling	IF*, WB^†^
Rhodopsin	Mouse	1:1000	Sigma-Aldrich	WB^†^
Red/green cone opsin	Rabbit	1:1000	Santa Cruz Biotechnology	WB^†^
Blue cone opsin	Rabbit	1:1000	Santa Cruz Biotechnology	WB^†^
Caspase-3	Rabbit	1:100	Santa Cruz Biotechnology	WB^†^
Beclin-1	Rabbit	1:1000	Cell Signaling	WB^†^
Atg5	Rabbit	1:1000	Cell Signaling	WB^†^
β-Actin	Mouse	1:2000	Sigma-Aldrich	WB^†^
GAPDH	Rabbit	1:5000	Sigma-Aldrich	WB^†^
Secondary Antibody
Anti-mouse Alexa Fluo 488	Goat	1:500	Molecular Probes	IF*
Anti-goat Alexa Fluo 488	Donkey	1:500	Molecular Probes	IF*
Anti-mouse Rhodamin RedX	Goat	1:1000	Jackson Immuno Research	IF*
Anti-rabbit Rhodamin RedX	Goat	1:1000	Jackson Immuno Research	IF*
Anti-mouse HRP	Goat	1:10000	Millipore	WB^†^
Anti-rabbit HRP	Goat	1:10000	Millipore	WB^†^
Anti-rabbit HRP	Goat	1:2000	Cell Signaling	WB^†^

*IF: Immunofluorescence; ^†^WB: Western blot.

### TUNEL Assay

Terminal deoxynucleotidyl transferase dUTP nick end labeling (TUNEL) staining was performed according to the manufacturer’s protocols for frozen sections (Promega, Milan, Italy). Sections pre-incubated with DNase were used as positive control. Sections from a retinal degeneration mouse (rd10 mutant; Gargini et al., 2007) known to contain apoptotic bodies were also used as positive controls. Nuclear staining was obtained with Hoechst (Life Technologies, Carlsbad, CA, USA). Images were taken with a Zeiss Apotome (Carl Zeiss, Oberkochen, Germany) microscope using a 16× lens. Individual images were digitally stitched together to reconstruct the entire section using Photoshop (Adobe, San Jose, CA, USA).

### Retinal Thickness Analysis

Cryosections where cell nuclei were labeled by 1 mM bis-benzimide were used for analysis. Images were acquired on a Zeiss Axiophot (Carl Zeiss) microscope using a 40× dry objective. Images were taken from dorsal and ventral retina, at distances between 250 and 500 μm (central retina) and higher than 500 μm (peripheral retina) from the optic nerve head.

For each section, eight different locations (field size: 290 × 320 μm) were measured and averaged. At least three sections per each retina were measured.

### Blood Vessels

The blood vessel complexity was estimated, on retinal whole mounts prepared as described elsewhere (Barone et al., 2012). Blood vessels were stained by incubating retinal whole mount for 3 days in FITC-conjugated Griffonia Simplicifolia (1:50 in artificial cerebro-spinal fluid and 0.1% Triton X-100, Sigma Aldrich) and examined with a Leica TCS SL confocal microscope. For each retina eight regularly spaced fields were sampled (937.5 × 937.5 μm in size). Sampled fields systematically covered central and peripheral areas of the four quadrants (Dorsal, Ventral, Nasal and Temporal). Within each field, three blood vessel plexa (superficial, intermediate and deep), were individually acquired along the *z*-axis at 1 μm intervals. Average intensity projection images of each plexa were used for morphometric analysis.

To calculate vascular area, the auto-threshold function of Metamorph software (Molecular Devices, LLC, USA) was chosen to identify vessels from background. Data from all quadrants of each retina were averaged, then values of each retina were used for plotting and statistical comparison. To calculate vascular complexity, each image was binarized using the Fiji’s threshold function (Schindelin et al., 2012), then intersected with a 50 × 50 μm mesh grid. Finally, intersection points in the resulting image were counted using the Fiji plugin “3D Objects counter” (Bolte and Cordelières, 2006) and expressed as number of intersections per mm^2^. Data from all quadrants of each retina were averaged, then values of each retina were used for plotting and statistical comparison.

### Electroretinogram (ERG)

The general procedure for animal preparation, anesthesia, ERG recording, light stimulation and data analysis has been previously described in detail (Della Santina et al., 2012). Following 4, 8 and 12 weeks of diabetes, control and diabetic mice were tested for scotopic and photopic ERG. For scotopic ERG recordings, mice were presented with a single flash of increasing intensity (2.19 × 10^−4^ to 83.7 cd*s/m^2^, 0.6 log units steps), each repeated five times, with an inter-stimulus interval ranging from 20 s for dim flashes to 1 min for the brightest flashes. Five ERG traces at each flash luminance were averaged. Isolated cone components were obtained by superimposing the test flashes (0.016 to 377 cd*s/m^2^), on a steady background of saturating intensity for rods (30 cd/m^2^) after at least 15 min from background onset. Oscillatory potentials (OPs) were also measured in both scotopic and photopic conditions. OPs were extracted digitally by using a fifth-order Butterworth filter as described by others (Hancock and Kraft, 2004; Lei et al., 2006). Peak amplitude and implicit time of each OP (OP1–OP4) were measured.

### Soluble and Insoluble Protein Separation and Western Blot Analysis

Detergent-insoluble protein aggregates were isolated according to Johnston et al. (1998). Retinas were lysed in 50 μl immunoprecipitation buffer (IPB, 10 mM Tris-HCl, 5 mM EDTA, 1% NP-40, 0.5% Na-deoxycholate, 150 mM NaCl, protease inhibitors, pH = 7.5) and incubated on ice for 30 min. Lysates were centrifuged for 15 min at 13,000 rpm then supernatants were collected (soluble proteins). The insoluble material was incubated in 50 μl of 10 mM Tris-HCl, 1% SDS for 10 min at room temperature. Following to the addiction of 50 μl IPB, pellets were sonicated for 20 s and the total protein content was quantified by Bradford method.

To assess rhodopsin, cone-opsin and apoptosis markers, 50 μg of soluble protein were electrophoresed on a sodium dodecyl sulfate (SDS)-polyacrylamide gel (12% for opsins, 15% for apoptosis markers). Proteins were transferred to polyvinylidene fluoride membrane (Immobilon-P Transfer membrane 0.45 μm, Millipore, Darmstadt, Germany) using a transfer buffer (25 mM Tris-HCl, pH = 8.3, 192 mM glycine, 20% methanol). To detect the presence of autophagy markers, 20 μg of insoluble proteins were loaded on 4–20% precast SDS-polyacrilamide gels. Proteins were blotted on nitrocellulose membrane (Sigma-Aldrich, 0.22 μm pore size).

For all cases, the protein blot was blocked for 1 h at room temperature with blocking solution (5% non-fat dried milk and 0.1% Tween-20 in 20 mM Tris-HCl, 500 mM NaCl, pH = 8). The membrane was then incubated overnight at 4°C with primary antibodies (Table 1) diluted in blocking buffer. Horseradish peroxidase (HRP)-conjugated secondary antibodies (Table 1) were incubated 2 h at room temperature. Bands were visualized using a chemo luminescence kit (Santa Cruz) and quantified by optical densitometry. Images were acquired using ImageQuant LAS4010 (GE Healthcare, Buckingamshire, UK). On the same blots, protein contents were normalized to the amounts of β-actin or Glyceraldehyde 3-phosphate dehydrogenase (GAPDH, Sigma-Aldrich).

### Statistical Analysis

Statistical comparisons for ERG, western blot, and blood vessel analysis were performed with analysis of variance (ANOVA) one-way or two-way test followed by Bonferroni-corrected *t*-test using Origin Lab 8.0 software (Microcal, Northampton, MA, USA).

## Results

Three groups of diabetic mice were separately tested 4, 8 and 12 weeks after a single dose of STZ i.p. Littermate mice that received a single dose of only the vehicle served as control groups. Table 2 shows the average serum glucose levels for all experimental animals at 4, 8 and 12 weeks following induction of diabetes. The starting body weight of control and diabetic mice was not different (*P* = 0.73, ANOVA). The weight of control mice increased constantly with time (+1 g/week), while that of diabetics either remained constant or decreased slightly (−0.56 g/week).

**Table 2:** **Blood glucose concentration**.

	4th Week		8th Week		12th Week	
Glycemia (mmol/l)	Vehicle	STZ	Vehicle	STZ	Vehicle	STZ
Before STZ	6.9 ± 0.3	8.9 ± 0.5	7.5 ± 0.4	8.3 ± 0.5	7.2 ± 0.4	9.3 ± 0.5
End of treatment	10.2 ± 0.4	28.4 ± 1.4	9.4 ± 0.6	30.5 ± 1.2	7.5 ± 0.4	27.9 ± 1.5
Variation Δ	3.3 ± 0.7	19.5 ± 1.9	1.9 ± 1.0	22.2 ± 1.7	0.3 ± 0.8	18.6 ± 2.0
P (*t*-test)	1.34 × 10^−9^		1.52 × 10^−12^		2.56 × 10^−10^	

*Glycaemia levels in vehicle- and STZ-injected mice used in the study. Data are expressed as average ± SEM. Statistical comparisons using independent two-sample t-test*.

**Figure 1:**
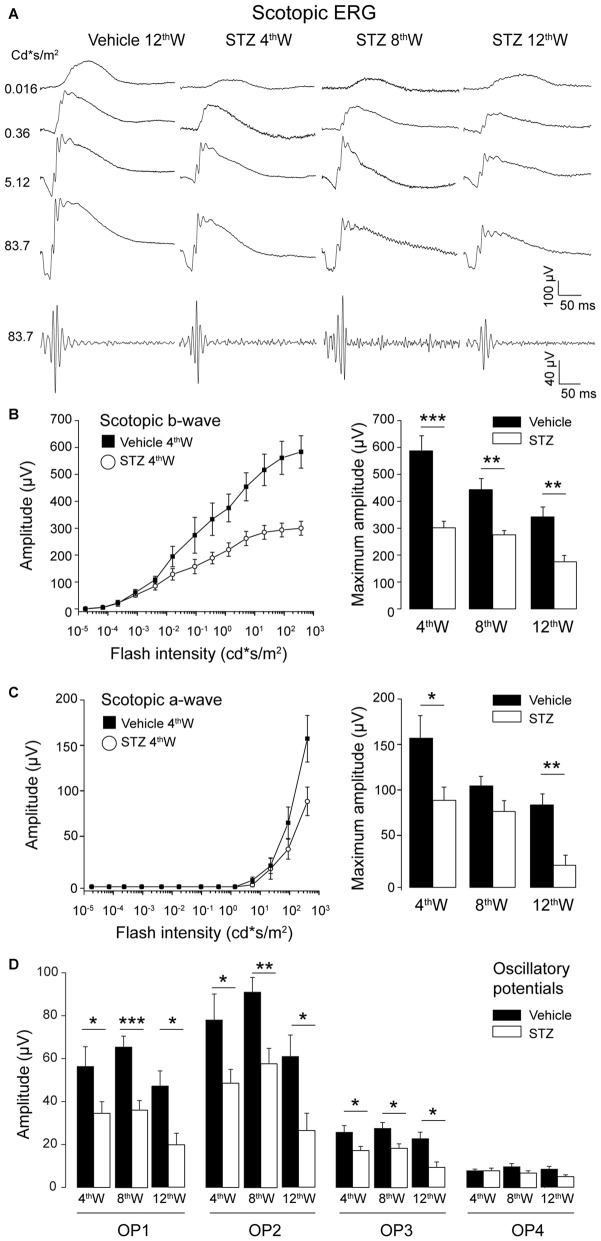
**Early impairment of outer and inner retinal function in hyperglycemic mice. (A)** Representative scotopic flash electroretinogram (ERG) responses from control and hyperglycemic mice 4, 8, 12 weeks after injection. Bottom traces: OPs filtered from the ERG response to the brightest flash. **(B,C)** Left: scotopic b-wave **(B)** and a-wave **(C)** amplitude as a function of flash intensity from 4 weeks vehicle- and STZ-injected mice. Right histograms: maximum b-wave **(B)** and a-wave **(C)** amplitude measured in response to the brightest flash (377 cd*s/m^2^) for control and hyperglycemic mice. **(D)** Average amplitude of oscillatory potentials (OP1 ÷ OP4) extracted from ERG response to the brightest flash (377 cd*s/m^2^). Values expressed as average ± SEM. Statistical analysis (*t*-test) *P < 0.05, **P < 0.01, ***P < 0.001. Number of vehicle- vs. STS-injected animals: 4th week: *N* = 7 vs. 13. 8th week: *N* = 6 vs. 9; 12th week: *N* = 6 vs. 7.

We recorded the ERG response in control and diabetic mice at different times following STZ injection. Figure 1A shows representative scotopic ERG responses to flashes of light of increasing luminance at different times of the disease progression. Traces at the bottom of Figure 1A are the OPs isolated from responses to the brightest flash. As shown in Figure 1B, the amplitude of the scotopic b-wave was reduced starting from 4 weeks after diabetes induction (Figure 1B, left plot, *P* = 8.8 × 10^−16^, two way ANOVA) and remained significantly lower than in control mice at all ages under examination (Figure 1B, histograms). In parallel, scotopic a-wave amplitude in diabetic mice was significantly reduced compared to control mice starting from 4 weeks after induction of diabetes (Figure 1C, *P* = 0.02, two-way ANOVA). Only a small reduction in time of the ERG amplitude was observed in the control groups across time while, at any given time point, significant difference of the scotopic ERG amplitude was observed between diabetic and control mice.

Amplitude and kinetics of the OPs (OP1, 2 and 3) was also affected in diabetic mice starting 4 weeks after injection of STZ: the amplitude decreased and implicit time increased in diabetic mice compared to control (Figure 1D, OP1: *P* = 0.22; OP2: *P* = 0.12; OP3: *P* = 0.07; OP4: *P* = 0.093, *t*-test). In order to investigate where the functional changes originate, we analyzed the b-wave vs. a-wave ratio. The average ratio remained constant at all time points analyzed (4 weeks: 16.05 ± 4.56; 8 weeks: 11.27 ± 3.00; 12 weeks: 12.58 ± 3.67; ANOVA one-way *P* = 0.69), suggesting that the early functional changes experienced by diabetic mice can be explained primarily by the damage inflicted to their rod photoreceptors.

**Figure 2:**
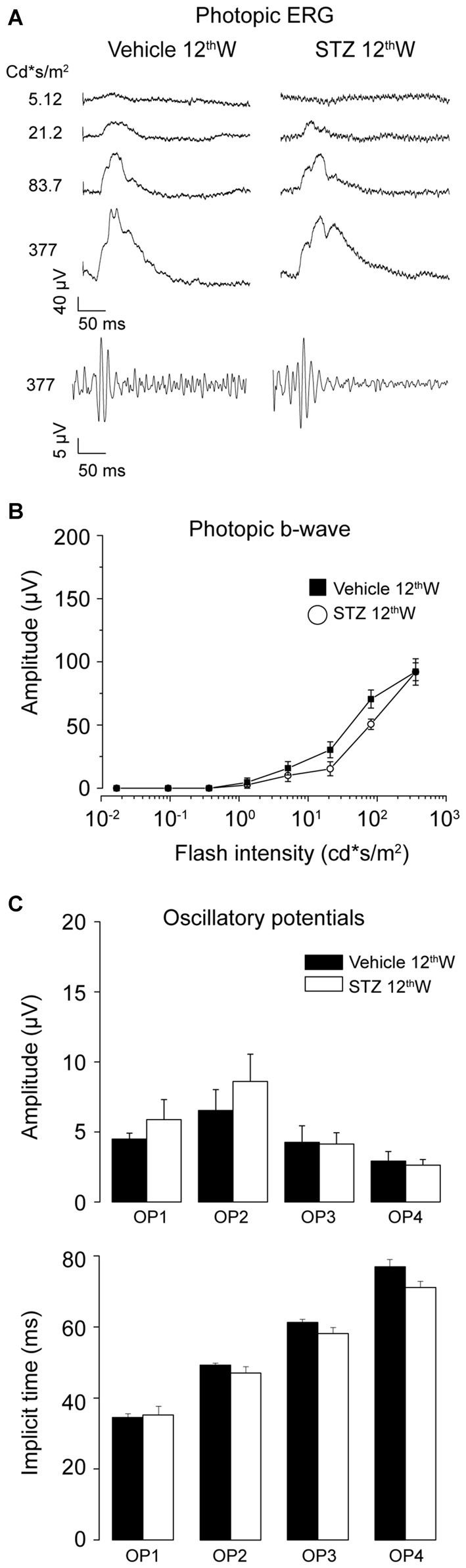
**Photopic ERG is not altered in hyperglycemic mice. (A)** Representative photopic flash ERG responses from control and hyperglycemic mice 12 weeks after injection. Bottom traces: OPs extracted from the ERG response to the brightest flash. **(B)** Left: photopic b-wave amplitude as a function of delivered flash intensity for vehicle-and STZ-injected mice, 12 weeks after injection. **(C)** Average amplitude (top) and implicit time (bottom) of oscillatory potentials (OP1 ÷ OP4) extracted from ERG response to the brightest flash (377 cd*s/m^2^). Values expressed as average ± SEM. Vehicle-injected *N* = 6, STZ, -injected *N* = 7 animals. Statistical analysis of vehicle vs. diabetic mice (*t*-test). Amplitude OP1–OP4: *P* = 0.34; 0.41; 0.94; 0.75. Implicit Time: OP1–OP4 : *P* = 0.78; 0.26; 0.12; 0.04.

We also recorded photopic ERG responses from the same animals (Figure 2). Figure 2A shows representative photopic ERG waveforms, and relative OPs, at increasing light intensity levels for mice 12 weeks from diabetes induction. As shown in Figure 2B, the amplitude of the photopic b-wave did not change up to 12 weeks following diabetes induction compared to control (*P* = 0.16, *t*-test). The amplitude and kinetics of the photopic OPs were not altered in diabetic mice compared to controls (Figure 2C). These results indicate that outer retina changes in diabetic mice mainly concern the rod pathway. It must be emphasized that impaired functionality always preceded the morphological signs of damage.

**Figure 3:**
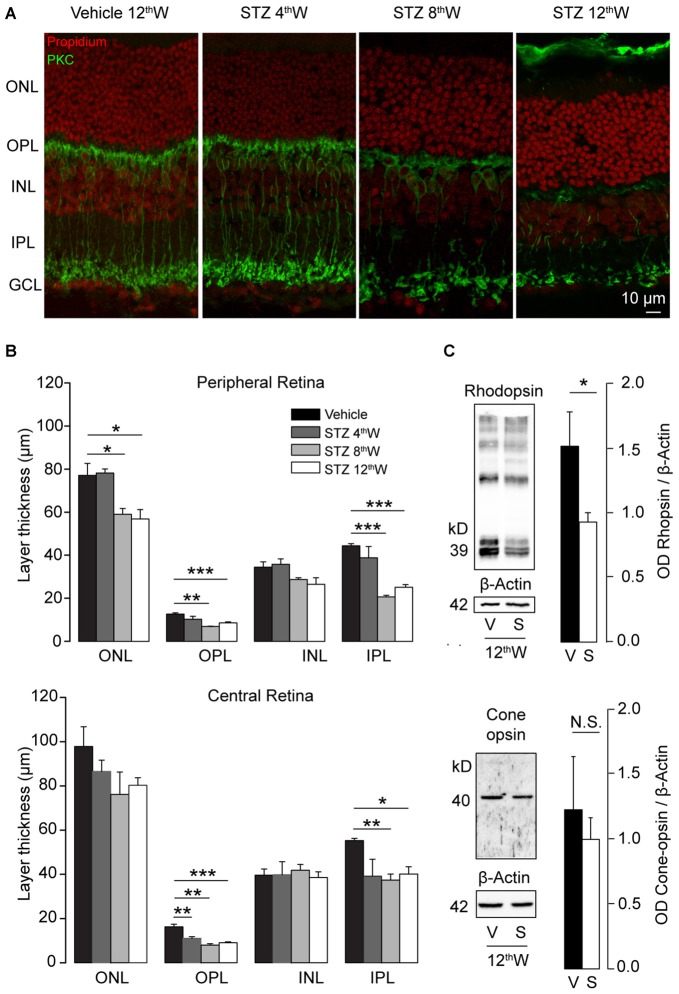
**Reduction of photoreceptors and retinal synaptic layers in hyperglycemic mice. (A)** Retinal sections from vehicle-injected and STZ-injected mice at 4, 8 and 12 weeks after induction of hyperglycemia. Nuclear layers (red) and rod bipolar cells (green) are labeled. **(B)** Retinal layer thickness in control and hyperglycemic mice. Top histograms: thickness in the peripheral retina for nuclear (ONL, INL) and synaptic layers (OPL, IPL). Bottom histograms: layer thickness in the central retina for the same regions. *N* = 3 animals for each condition. **(C)** Western blot analysis of rhodopsin and cone-opsin content in retinas of control and hyperglycemic mice, collected 12 weeks after injection. Left images: example western blots. Right histograms: optical density ratio opsin/β-actin in control and hyperglycemic mice. *N* = 6 animals for each condition. Values expressed as average ± SEM. Statistical analysis (*t*-test) *P < 0.05, **P < 0.01, ***P < 0.001.

**Figure 4:**
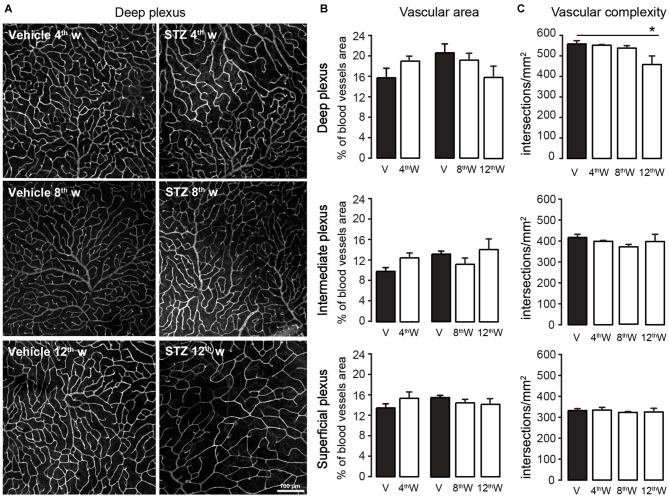
**Retinal blood vessels area and complexity. (A)** Representative images of retinal blood vessel in the deep plexus in vehicle-injected and STZ-injected mice at 4, 8 and 12 weeks from injection. Blood vessels were stained with Lectin. **(B)** Quantification of relative vascular area, expressed as percentage of area in the plexus taken by blood vessels, within the deep (top plot) intermediate (middle) and superficial (bottom) plexa. **(C)** Quantification of vascular complexity, expressed as number of intersections between blood vessels per mm^2^, within the deep (top plot), intermediate (middle) and superficial (bottom) plexa. Values expressed as average ± SEM. Statistical analysis (*t*-test) *P < 0.05. Number of vehicle- vs. STZ-injected animals 4th week: *N* = 3 vs. 5; 8th week: *N* = 4 vs. 5; 12th week: *N* = 3 vs. 6.

The panels of Figure 3A show a vertical section of retinas from control (12 weeks post vehicle-injections) and diabetic mice (4, 8 and 12 weeks post STZ-injections) where reduction in the total thickness of the retina can be observed. Furthermore, specific staining of rod-bipolar cells (PKCα, green) shows that, starting from 8 weeks after induction of diabetes, there was a strong regression of dendrites, axons and synaptic terminals. In the central retina, thickness was reduced 4 weeks after diabetes induction for the outer plexiform layer (OPL) and after 8 weeks also for the inner plexiform layer (IPL, Figure 3B, lower panel). On the other hand, thickness of synaptic layers in the peripheral retina decreased since 8 weeks from STZ injection (Figure 3B, upper panel), supporting a center-to-periphery gradient of synaptic degeneration. Because no significant differences were observed among animals of the control groups all vehicle measurements are shown in Figure 3B as pooled data in the same column (*P* = 0.25 for layers in central retina; *P* = 0.125 for periphery outer and inner nuclear layers, ONL and INL; *P* = 0.063 for periphery OPL and IPL, one-sample Wilcoxon signed paired ranks test). Western blot analysis of retinal opsins (Figure 3C) showed a significant reduction of rhodopsin expression (*P* = 0.017, unpaired *t*-test) but not of cone-opsins (*P* = 0.34, unpaired *t*-test), providing additional support to the notion that the initial damage caused by DR in the outer retina primary affects rods but not cones. In order to test whether the damage of the neural retina is correlated with vascular changes we also examined morphology of retinal blood vessels (Figure 4). Area (Figure 4B) and complexity of the vessels (Figure 4C) was determined for each of the three vascular plexa (see “Materials and Methods” Section).

The vascular area in each plexus was similar in control and diabetic animals (Figure 4B; for 4, 8 and 12 week respectively; deep plexus: *P* = 0.14; 0.54; 0.11; intermediate plexus: *P* = 0.10; 0.15; 0.67; superficial plexus: *P* = 0.33; 0.21; 0.26). Because in the time frame of 8–12 months control animals had similar blood vessels complexity (*P* = 0.75, one-way ANOVA) data were pooled. The complexity of deep plexus vessels, supplying photoreceptors and bipolar cells, was significantly reduced in diabetic mice only 12 weeks post-injection (Figure 4C). In addition, no signs of neovascularization were detected throughout the period of observation. These results suggest that the damage to the neural retina in the early stages of DR cannot be attributed to a vascular injury.

**Figure 5:**
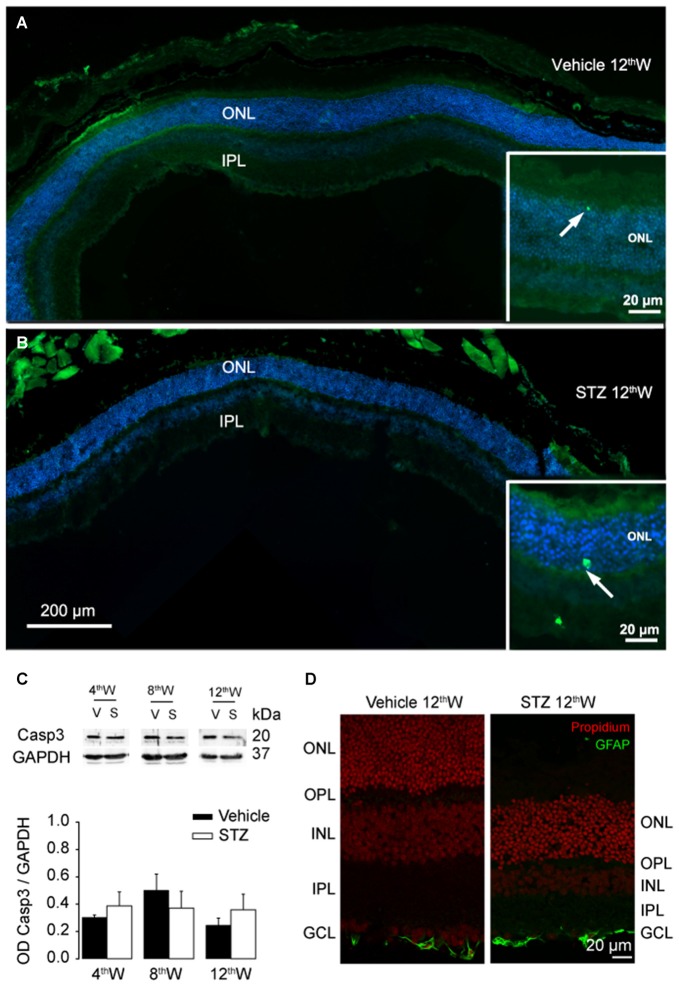
**Lack of apoptosis activation in photoreceptors of hyperglycemic mice. (A,B)** Representative retinal sections from control and hyperglycemic mice, 12 weeks after injection. Apoptotic cells (green) labeled with TUNEL staining, cellular nuclei labeled with Hoechst (blue). Insets: magnification of the area where sporadic apoptotic cells were found. **(C)** Top: representative western blots labeled against the activated form of the apoptosis effector Caspase-3 and the control protein GAPDH. Bottom histograms: average ratio of the optical density of Caspase-3 and GAPDH after 4, 8, 12 weeks from injection. Values expressed as average ± SEM. Statistical analysis in vehicle vs. diabetic mice (*t*-test): 4th week: *P* = 0.54; 8th week: *P* = 0.46; 12th week: *P* = 0.29. *N* = 6 animals per each condition. **(D)** Retinal sections from control and hyperglycemic mice 12 weeks after injection. Cellular nuclei are labeled with propidium iodide (red). Activated Müller glia was labeled with anti-GFAP antibody (green).

We then investigated whether rod photoreceptors die by apoptosis, as reported for retinal ganglion cells (Barber et al., 1998; Park et al., 2003; Martin et al., 2004). Surprisingly, hardly any sign of apoptosis was detected in STZ-injected mice within the time frame of our analysis. Representative retinal sections stained for apoptotic cells with TUNEL (Figures 5A,B, 12 weeks after injection) show only rare apoptotic figures in both diabetic and control retinas. Furthermore, expression of activated Caspase-3 (Figure 5C) was not increased in diabetic mice with respect to control (4 weeks: *P* = 0.54; 8 weeks: *P* = 0.46; 12 weeks: *P* = 0.29). We also found that in contrast to other forms of retinal degenerations, such as retinitis pigmentosa (Gargini et al., 2007), Müller glia was not activated (Figure 5D, 12 weeks). Together, these data suggest that rod photoreceptors death does not involve apoptosis in the early stage of STZ-induced DR. A possible alternative pathway leading to rod degeneration in DR is thus the dysregulation of autophagic flow.

**Figure 6:**
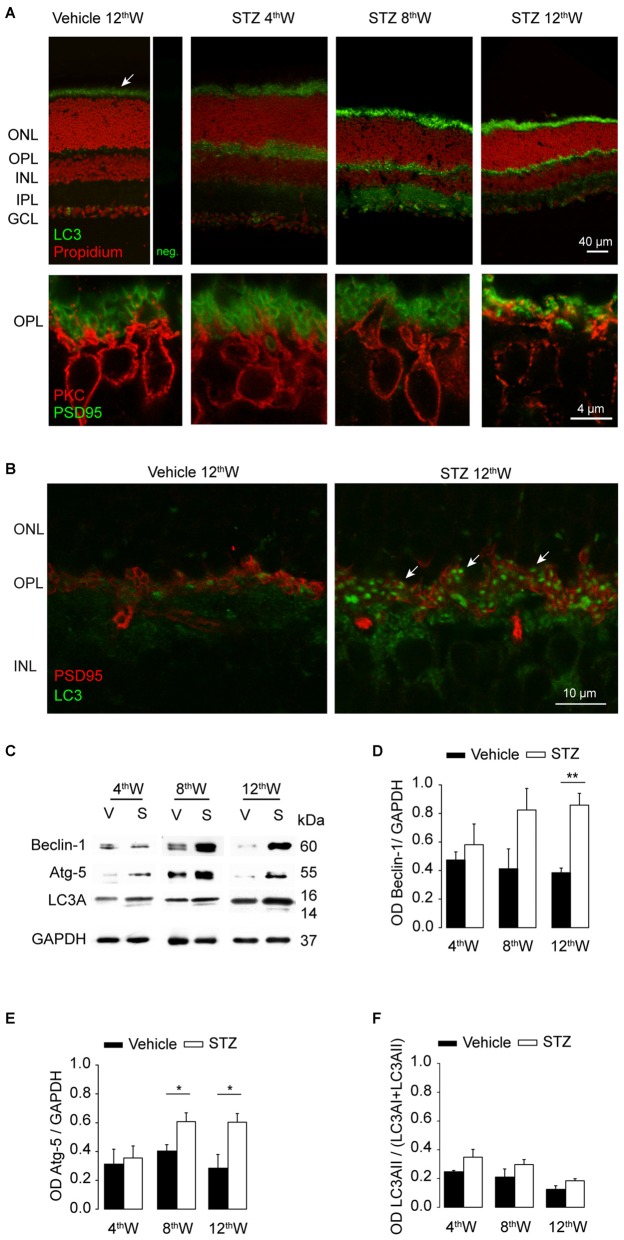
**Upregulation of autophagic proteins in hyperglycemic mice. (A)** Top row: LC3 localization (green) in control and in STZ-injected retinas, nuclei in red (propidium iodide). Top left panel shows control retina next to negative control labeling with secondary antibody (neg.). The arrow indicates autophagy normally taking place during photoreceptor outer segments renewal. Bottom row: photoreceptor presynaptic protein PSD95 (green) and rod bipolar cells (red) labeled at the level of OPL. **(B)** Double-staining with PSD95 (red) and LC3 (green) at the level of OPL in retinas from control and diabetic mice, 12 weeks from injection. Arrows indicate in STZ-treated retina the punctate accumulation of LC3 at the level of OPL. **(C)** Representative western blot experiments. Autophagic markers Beclin-1, Atg-5 and light chain 3A (LC3A) as well as control GAPDH were assayed at 4, 8 and 12 weeks from injection. **(D,E)** Quantification of the average ratio between autophagic proteins Beclin-1 **(D)** Atg-5 **(E)** and the control protein GAPDH. **(F)** Quantification of the fraction of LC3AII (activated isoform) over total LC3A content at 4, 8 and 12 weeks from injections. Values expressed as average ± SEM. Statistical analysis (*t*-test) *P < 0.05, **P < 0.01, ***P < 0.001. *N* = 6 animals for each condition.

We thus monitored three markers of autophagy: Beclin-1, Atg5 and the microtubule-associated protein 1A/1B-light chain 3A (LC3A) that play critical roles in the nucleation, expansion and conjugation steps of autophagosome formation, respectively. LC3A undergoes cleavage and lipidation to LC3A-II (Larsen and Sulzer, 2002; Mehrpour et al., 2010) becoming a component of the mature autophagosome (Mizushima and Yoshimori, 2007). In control retina, LC3A (Figure 6A, top panel) was mainly present in photoreceptor outer segments, normally involved in the process of the discs shedding and renewal (Remé and Sulser, 1977; Remé et al., 1986). In diabetic mice, LC3A (Figure 6A, top panels) labeling was stronger in photoreceptors as well as in synaptic layers compared to control retinas. In parallel, the architecture of photoreceptor synaptic terminals and rod bipolar cell dendrites was progressively disrupted (Figure 6A, bottom panels). In parallel to changes observed in synaptic layers, LC3A labeling increases also in the ganglion cell layer, indicating increased autophagic activity of retinal ganglion cells and/or displaced amacrine cells. LC3A assumed a punctuate distribution at the level of OPL in diabetic mice (Figure 6B), next to photoreceptors synaptic terminals indicated by the presynaptic protein PSD95.

Retinal levels for Beclin-1, Atg5 and LC3A at each temporal point were investigated in both vehicle- and STZ-injected mice (Figure 6C). Quantification of these proteins (Figures 6D–F) revealed an increase in both Atg5 and Beclin-1 in the retinas of diabetic mice compared to control (*P* = 0.03 and 0.006, respectively, 12 weeks after diabetes induction, *t*-test). Quantification of the fraction of LC3A-II over total LC3A (Figure 6F) showed a tendency to increase in diabetic retinas but not to a significant level, possibly due to the presence of a strong physiological autophagic flux within photoreceptor outer segments.

## Discussion

This study examined the electrophysiological, morphological and biochemical changes occurring in the mouse retina as a result of STZ-induced diabetes. Overall, our results show that one of the earliest signs of the disease is a selective alteration of the rod system, while the cone pathway is largely spared. Rods thus appear to be particularly vulnerable to the effects of diabetes, possibly due to their high metabolic demands (Demontis et al., 1997). Our results showing reduction of scotopic a- and b- wave are in agreement with data in diabetic rat models previously reported by other authors (Shirao and Kawasaki, 1998; Li et al., 2002; Phipps et al., 2004, 2006; Aung et al., 2013). We have extended the electrophysiological analysis to the OP component of ERG to show that it is altered in diabetic mice. Rod dysfunction is correlated with thinning of synaptic layers and altered morphology of second order neurons, in accordance with evidence found in a genetic mouse model (Ins2^Akita^) of DR (Hombrebueno et al., 2014). Within the time frame of our analysis, vascular permeability was neither significantly altered nor did we observe any signs of neovascularization. Complexity of blood vessels supplying photoreceptors and bipolar cells is significantly reduced only 12 weeks from diabetes induction, well after the first signs of functional and morphological alterations.

No strong evidence for apoptosis was observed in the mouse retina over the duration of diabetes examined here. Our results are consistent with results in two rat models of diabetes (Énzsöly et al., 2014). Although one cannot exclude the possibility of undetected short lasting apoptotic events, functional and morphological data point to a continuous progression of the damage during the first 12 weeks after STZ injection. Conversely, a substantial increase of autophagic activity was shown in diabetic retinas using a variety of tests. Autophagy is generally regarded as a survival mechanism, and its deregulation has been linked to non-apoptotic cell death (Mehrpour et al., 2010; Nikoletopoulou et al., 2015). Here, we observe an increased expression of autophagic markers and in particular the staining of LC3A increases mainly in the OPL where an impairment of both pre- and post-synaptic proteins is apparent. Down-regulation of photoreceptor presynaptic proteins has been observed in other rodent models of diabetes (Park et al., 2003; Ozawa et al., 2011; Hombrebueno et al., 2014) as well as in patients with mild DR (Varga et al., 2015). Multiple evidence from cultured retinal explants suggests that high glucose exposure is sufficient to cause altered expression of key synaptic components such as vesicular GABA transporter (Baptista et al., 2015), purinergic receptors (Vindeirinho et al., 2013) and extracellular ATP levels (Costa et al., 2009), thus making the synaptic layers of the retina particularly prone to alterations occurring in early phases of DR. We therefore propose that the synaptic contacts in the outer retina undergo increased autophagy in response to diabetes. Up-regulation of autophagy occurs within the same time frame of outer retina damage and thus possibly represents the process leading to photoreceptor death in the early phase of DR. Recently other studies reported the up-regulation of autophagic pathway in the progression of neurodegenerative diseases such as glaucoma (Munemasa and Kitaoka, 2015; Wei et al., 2015) and suggested that the decrease of the autophagic pathway triggers degeneration of the retinal pigment epithelium (RPE; Yao et al., 2015).

It is perhaps interesting to note that deregulation of autophagy in diabetic retinas affects rods, a type of visual cells in which this process is particularly active in physiological conditions because of outer segment disc turnover and shedding (Remé et al., 1986). The identification of dysregulated autophagy affecting retinal neurons and their synaptic connections in the early stage of DR may open interesting perspectives for novel therapeutic strategies targeting key proteins of the autophagic pathway to restore the normal condition of this physiological process. Recent evidence in cell cultures supports this idea by showing that inhibition of autophagy may protect retinal cells from high glucose stress (Shi et al., 2015).

Collectively, our data show for the first time that, in addition to known apoptotic processes, autophagic pathway is probably involved in neuronal retinal degeneration. The present results may be useful for the exploration of new cell death pathways involved in the progression of DR and new targets could be evaluated in order to protect retinal neurons from damage.

## Author Contributions

IP, EN, LDS performed the experiments and analyzed data. IP, LDS, ES, LC designed the experiments, contributed to discussion. LDS, CG, LC, IP wrote the manuscript.

## Conflict of Interest Statement

The authors declare that the research was conducted in the absence of any commercial or financial relationships that could be construed as a potential conflict of interest.
